# FASN Knockdown Inhibited Anoikis Resistance of Gastric Cancer Cells via P-ERK1/2/Bcl-xL Pathway

**DOI:** 10.1155/2021/6674204

**Published:** 2021-08-19

**Authors:** Li Yu, Xin Wang, Yao Du, Xiaowen Zhang, Yunzhi Ling

**Affiliations:** ^1^Center of Clinical Laboratory Diagnostics, Bengbu Medical College, Bengbu City, Anhui Province 233030, China; ^2^Department of Anesthesiology, The First Affiliated Hospital of Bengbu Medical College, Bengbu City, Anhui Province 233000, China

## Abstract

Anoikis resistance (AR) is a crucial step in tumor metastasis. The overexpression of fatty acid synthase (FASN) is not only related to the AR of osteosarcoma cells, but also evidenced on gastric cancer (GC). This study investigated the role of FASN in the AR of GC cells. Plates coated with poly-HEMA were used for the culture of cells with AR. Small interfering RNA targeting FASN (siFASN) was transfected into MNK-45 and AGS cells. The number and apoptosis of cells were assessed by a hemacytometer and Annexin-V-FITC/PI assay, respectively. Aggregated cells and colony numbers were manually counted under a microscope. The migration and invasion rates were measured via wound healing and Transwell invasion assays, respectively. The levels of FASN, phosphorylated (p)-ERK1/2, ERK1/2 and Bcl-xL were detected through western blot or quantitative reverse transcription-polymerase chain reaction (qRT-PCR). The results showed that the cell numbers of *MNK-45* and *AGS* were increased while that of *GES-1* cell was decreased during the culture in suspension. A higher apoptosis rate and a smaller number of aggregated cells were observed in *GES-1* cells in comparison with *MNK-45* and *AGS* cells. A larger colony number, greater migration and invasion rates, and higher mRNA and protein expressions of FASN were presented in the AR group compared with the control group. Cells transfected with siFASN possessed lower migration and invasion rates, reduced expressions of FASN mRNA and protein, p-ERK1/2 and Bcl-xL, and induced a significantly declined ratio of p-ERK1/2 to ERK1/2. These findings suggest that down-regulation of FASN suppresses the AR of GC cells, which may be related to the inhibition of p-ERK1/2/Bcl-xL pathway.

## 1. Introduction

Gastric cancer (GC) is one of the most common malignant tumors in the world and ranks the third leading cause of cancer-related mortality, following lung and liver cancer [1]. Despite the decreased prevalence owing to the progresses made in medical technology, the prognosis of GC still remains poor, with a 5-year survival rate of less than 30% [2]. And metastasis is the main cause of deaths in patients with GC [3]. As a kind of programmed cell death which responds to the detachment of cells from the original extracellular matrix (ECM), anoikis can prevent cells from growth and reattachment in a different site, thus blocking the colonization of cells in distant organs. Unlike the normal cells, cancer cells show a resistance against anoikis, which contribute to the development and metastasis of tumors [4, 5]. Therefore, studies on the anoikis resistance (AR) of GC cells are of considerable significance for the prevention and treatment of metastasis in GC and the reduction on GC-induced mortality.

Fatty acid synthase (FASN) is a main enzyme participating in the formation of the newborn fat and is overexpressed in tissues of various tumors, such as prostate cancer and bone tumor [6–9]. Previous studies have proved an evidence on the interaction between FASN and the process of epithelial-mesenchymal transition (EMT) in GC [10], which reveals that FASN may be a promising therapeutic target for the anti-cancer therapy. However, studies on the detailed effect of FASN on GC cells with AR remain inadequate. Moreover, FASN was shown to mediate breast cancer through the regulation of ERK1/2 [11, 12]. Extracellular regulated protein kinases (ERK), including ERK1 and ERK2, can be activated into phosphorylated (p)-ERK so as to promote the multiplication and suppress the apoptosis in the cells. Previous studies also revealed the relation on the activation of ERK activation and the cancer and AR [13–15]. Bcl-xL, an anti-apoptotic member of the B-cell lymphoma-2 (Bcl-2) family, exerts a protective effect on cancer cells, including gastric cancer cells, as reported in previous publications [16–18]. Besides, the upregulation of FASN was proved to be associated with the AR of osteosarcoma cells via p-ERK1/2/Bcl-xL [19]. Consequently, we tried to explore whether FASN realizes its effect on AR via ERK1/2/Bcl-xL pathway in GC.

In this study, the anoikis resistant GC cells were cultured *in vitro*, the purpose of which was to explore the biological characteristics of AR. In addition, the role and molecular mechanism of FASN in GC cells with AR were also investigated *in vitro* by controlling variables, the results of which demonstrated that FASN mediated AR in GC cells through p-ERK1/2/Bcl-xL pathway, making FASN as a promising therapeutic target for the intervention and remedy of metastasis in GC.

## 2. Materials and Methods

### 2.1. Cell Culture

Normal gastric mucosa epithelial cell line GES-1 and GC cell lines MNK-45 and AGS were obtained from Shanghai Institutes for Biological Sciences (Shanghai, China) and were cultured in RPMI-1640 medium (E600028-0500, Sangon Biotech Co., Ltd., Shanghai, China) which was supplemented with 10% fetal bovine serum (FBS; E600001-0500, Sangon Biotech Co., Ltd., Shanghai, China), 1% L-glutamine (A600224-0500, Sangon Biotech Co., Ltd., Shanghai, China) and Benzylpenicillin/streptomycin (B540732-0010, Sangon Biotech Co., Ltd., Shanghai, China) at 37°C in an incubator containing 5% CO_2_.

### 2.2. Suspension Culture of Anoikis Resistant GC Cells

The adherent cells were trypsinized (A610681-0100, Sangon Biotech Co., Ltd., Shanghai, China) so as to obtain the single cell suspension and then seeded at 10^6^ per well in Costar ultra-low attachment 6-well plates (3471, Corning Incorporation, Corning, NY, USA). Poly-hydroxyethyl methacrylate (poly-HEMA, ST1582, Beyotime Biotechnology, Shanghai, China) was pre-dissolved in 95% ethanol (493511, Sigma-Aldrich, Ontario, Canada), and the solution was added into the culture plates to induce the anoikis in the cell. The cells were passaged every three days. After continuous passages, the anoikis resistant cells were selected and collected as AR group for biological characteristics analysis. Cells under adherent culture were used as the control group and received no treatment.

### 2.3. Suspension Culture and Detection

The adherent cells of the control and AR groups were trypsinized to the single cell suspension and then seeded at 10^6^ per well in costar ultra-low attachment 24-well plates (3473, Corning Incorporation, Corning, NY, USA). A hemacytometer (AMQAF1000, Thermo Fisher Scientific, Waltham, MA, USA) was used to count the numbers of cells on days 0, 2, 4 and 6. The morphology of cells was observed using a phase contrast microscope (magnification: 100x; DYS-810, Shanghai Dianying Optical Instrument Co., Ltd., Shanghai, China).

### 2.4. Cell Apoptosis Assay

After the culture in suspension for 24 hours (h), the apoptosis of adherent and detached cells was assessed by an Annexin-V-FITC Apoptosis Detection Kit with PI (E606336-0500, Sangon Biotech Co., Ltd., Shanghai, China). The cells were washed 3 times with PBS and then resuspended in binding buffer. Annexin V-FITC and PI were added to the solution containing cells in a dark room at room temperature for 15 minutes (min). The apoptosis rates were analyzed in a flow cytometer (CytoFLEX, Beckman Coulter, Brea, CA, USA).

### 2.5. Colony Formation Assay

Cells in logarithmic phase were trypsinized to single cell suspension and then seeded at 10^5^ per well in Costar ultra-low attachment 24-well plates for 7 days of suspension culture. Then the cell suspension was seeded into normal 24-well plates (J00240, Shanghai Jinan Biological Technology Co., Ltd., Shanghai, China) until the formation of colonies. After being fixed with 4% paraformaldehyde (E672002-0500, Sangon Biotech Co., Ltd., Shanghai, China) at room temperature for 15 min and stained with crystal violet (E607309-0100, Sangon Biotech Co., Ltd., Shanghai, China) for 15 min, the colonies were manually counted.

### 2.6. Wound Healing Assay

Cells were seeded in 6-well plates (J00660, Shanghai Jinan Biological Technology Co., Ltd., Shanghai, China) at a density of 5 × 10^6^ cells per well and cultured until 80-90% confluence. A pipette (Sangon Biotech Co., Ltd., Shanghai, China) tip was used to make a straight scratch which simulated as a wound. Cells were rinsed with PBS and the medium was replaced with serum-free medium at 37°C for 24 h. Images were observed and recorded at 0 and 24 h by a DYS-810 microscope (magnification: 100 x; Shanghai Dianying Optical Instrument Co., Ltd., Shanghai, China) and the distance for the migration was measured by Image J, version 1.48 (National Center for Biotechnology Information, Bethesda, MD, USA). Six visual fields were randomly selected from each room.

### 2.7. Transwell Invasion Assay

Cell invasion assay was performed in a 24-well Transwell chamber (3379, Corning Incorporation, Corning, NY, USA). The upper filters were pre-coated with Matrigel (354234, Corning Incorporation, Corning, NY, USA), and cells with serum-free medium were seeded into the upper chambers at a density of 4 × 10^5^ cell/ml. The medium in the lower chamber contained 10% FBS and functioned as a source of chemoattractants. After the incubation at 37°C for 24 h, cells that passed through the Matrigel-coated membrane were fixed with 4% paraformaldehyde at 4°C for 30 min and then stained with crystal violet at room temperature for 30 min. Cells in six randomly selected visual fields from each room were counted under a NEXCOPE-NIB-620 inverted biological microscope (magnification: 250x; Chensheng Optical Instrument Technology Co., Ltd., Shenzheng, China).

### 2.8. Transfection of FASN Silencing RNA

Cells in logarithmic phase were trypsinized, seeded in a 24-well plate at a density of 5 × 10^3^ cells per well and cultured until 30-50% confluence. To downregulate the expression of FASN, small interfering RNA targeting FASN (siFASN; siB111124103713-1-5, UGAGAAAGGUCGAAUUUGCCA, Guangzhou RiboBio Co., Ltd, Guangzhou, China) was transfected into the cells. Lipofectamine 2000 (11668019, Thermo Fisher Scientific, Waltham, MA, USA) was used during the 48 h transfection of siFASN and small interfering RNA of negative control (siNC; siN0000002-1-5, Guangzhou RiboBio Co., Ltd, Guangzhou, China).

### 2.9. Quantitative Reverse Transcription-Polymerase Chain Reaction (qRT-PCR)

Total RNA was extracted from cells using a TRIzol kit (B511321-0100, Sangon Biotech Co., Ltd., Shanghai, China). Reverse transcription was conducted to synthesize cDNA by using a PrimeScript RT Reagent Kit (638314, Takara Bio, Inc., Otsu, Japan). Sequences of the primers (Shanghai GenePharma Co., Ltd., Shanghai, China) were listed in Table 1. Then the cDNA was tracked using SYBR Green qPCR Supermix (1725270, Bio-Rad Laboratories, Inc., CA, USA) through a CFX384 RT-PCR cycler (Bio-Rad Laboratories, Inc., CA, USA) under a thermal cycling program containing 40 cycles of 95°C for 3 min, 95°C for 15 s, and 60°C for 15 s. The expression levels of relative FASN RNA were calculated on the basis of the mean *β*-actin expression levels in the representative sample. Data were analyzed using the relative quantification method 2^-*ΔΔ*CT^ [20].

### 2.10. Western Blot

Total protein was extracted from cells using radio immunoprecipitation assay (RIPA) lysis buffer (R0010-100 ml, Beijing Solarbio Science & Technology Co., Ltd., Beijing, China) with phenylmethanesulfonyl fluoride (PMSF) (P0100-100 ml, Beijing Solarbio Science & Technology Co., Ltd., Beijing, China), and then the protein was centrifuged at 12,000 × g at 4°C for 10 min to collect the supernatant. The concentration of total protein was determined by a Bradford assay kit (5000001, Bio-Rad Laboratories, Inc., CA, USA). Equal amounts of protein samples were separated by 10% SDS (A600485-0100, Sangon Biotech Co., Ltd., Shanghai, China) polyacrylamide gel electrophoresis (SDS-PAGE) and transferred to PVDF membranes (88585, Thermo Fisher Scientific, Waltham, MA, USA). The membranes were blocked in 5% bovine serum albumin (BSA; E661003-0100, Sangon Biotech Co., Ltd., Shanghai, China) for 1 h and then incubated overnight at 4°C with the antibodies including anti-FASN (ab128870, 1 : 10000; Abcam, Cambridge, MA, USA), anti-p-ERK1/2 (ab214362, 1 : 1000; Abcam, Cambridge, MA, USA), anti-ERK1/2 (ab184699, 1 : 10000; Abcam, Cambridge, MA, USA), anti-Bcl-x (ab32370, 1 : 1000; Abcam, Cambridge, MA, USA), and anti-*β*-actin (ab8226, 1 : 1000; Abcam, Cambridge, MA, USA). The next day, the membranes were removed and washed with Tris-buffered saline containing Tween (TBST; C520009-0001, Sangon Biotech Co., Ltd., Shanghai, China), followed by being incubated with corresponding secondary antibodies conjugated to Horseradish Peroxidase (HRP; ab6721, 1 : 5000; Abcam, Cambridge, MA, USA) for 1 h at room temperature and washed five times with TBST for 5 min each time. ECL reagent (32209, Thermo Fisher Scientific, Waltham, MA, USA) was added to the membranes for the visualization in a dark room at room temperature. The protein level was analyzed using Image J software, version 1.48 (National Institutes of Health, Bethesda, MD, USA).

### 2.11. Statistical Analysis

GraphPad Prism 8.0 (GraphPad Software Inc., San Diego, CA, USA) and SPSS 20.0 software (SPSS Inc., Chicago, IL, USA) were used to conduct the statistical analysis. Data were performed as the means ± standard. The statistical significance of differences between two groups was determined by independent-samples *t*-test. Comparisons among more than two groups were conducted using one-way ANOVA followed by Tukey's post hoc test. P <0.05 was considered to be a statistically significant difference.

## 3. Results

### 3.1. GC Cells Showed Stronger AR Compared with Normal Gastric Mucosa Epithelial Cells

**Figure 1:**
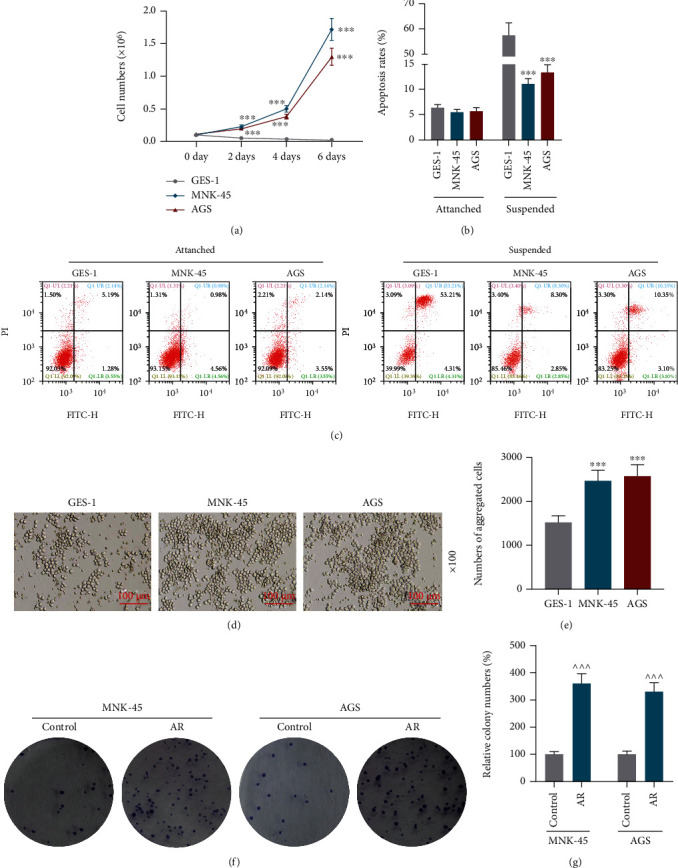
Gastric cancer cells have stronger anoikis resistance compared with normal gastric mucosa epithelial cells. (a) Numbers of *GES-1*, *MNK-45* and *AGS* cells were counted by a hemacytometer after suspension culture for 0, 2, 4, and 6 days. (b) Apoptosis rates of *GES-1*, *MNK-45* and *AGS* cells after 24-hour adherent culture or suspension culture were quantitated through cell apoptosis assay. (c) Cell apoptosis was evaluated using a flow cytometer and an Annexin-V-FITC Apoptosis Detection Kit with PI. (d) Pictures of the morphology of *GES-1*, *MNK-45* and *AGS* cells in suspension culture were taken under a microscope. (e) Numbers of aggregated *GES-1*, *MNK-45* and *AGS* cells in suspension culture were counted by microscopy. (f) Representative pictures of colonies in colony formation assay. (g) Relative cell colony numbers were counted manually. ^∗^*p* <0.05 vs. *GES-1* cells; ^∗∗∗^*p* <0.001 vs. *GES-1* cells and *^^^^^ p* <0.001 vs. control group. Data were performed as the means ± standard.

During the culture in suspension for 2, 4 and 6 days, the number of *GES-1* cells decreased while those of *MNK-45* and *AGS* cells increased significantly (Figure 1(a), *p*<0.001). *GES-1*, *MNK-45* and *AGS* cells were all attached cells, some of which died during the culture in suspension, and we found the apoptosis rate of the *GES-1* cells in suspension was higher than the attached *GES-1* cells. And the apoptosis rate of normal gastric mucosa epithelial cells was higher than GC cells in both attached and suspended condition (Figures 1(b), 1(c)*, p* < 0.05, *p* <0.001). In suspension culture, a multicellular aggregate is more resistant to anoikis than single suspension cells [21]. As suggested by the results of the morphology on the cell, which was observed by microscopy,, *MNK-45* and *AGS* cells formed larger clusters and more aggregated cells when compared with *GES-1* cells (Figures 1(d), 1(e)*, p* <0.001).

### 3.2. AR Promoted GC Cell Multiplication, Migration and Invasion and FASN Was Associated with AR

**Figure 2:**
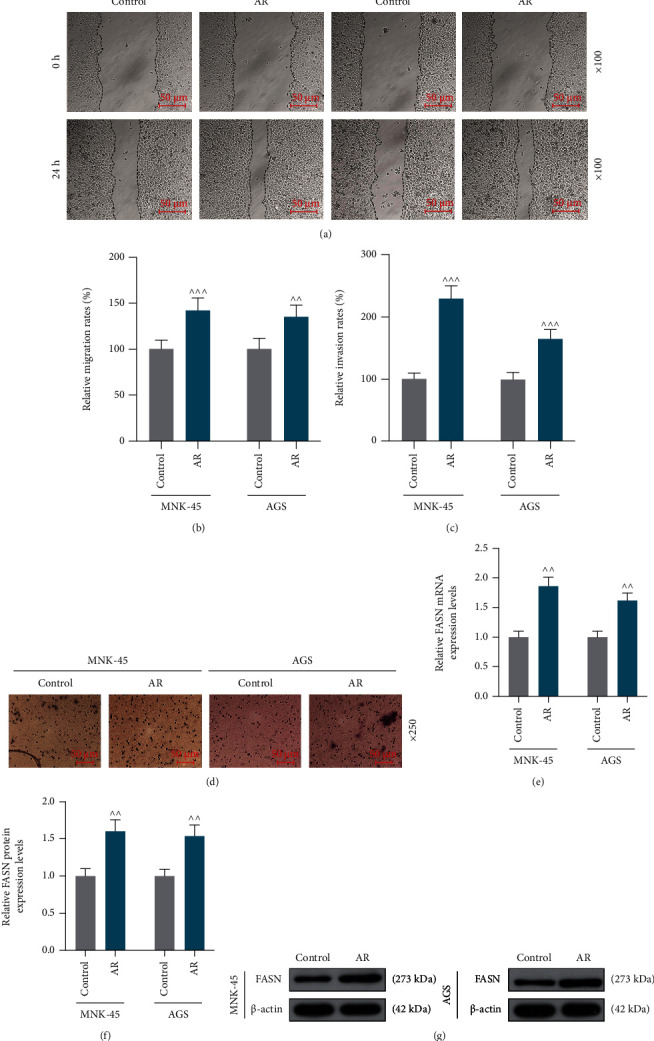
Anoikis resistance (AR) promoted gastric cancer cell migration and invasion, and fatty acid synthase (FASN) was related to AR. (a) Representative images of the migration of *MNK-45* and *AGS* cell in the AR and control groups determined by wound healing assay. (b) Relative migration rates of *MNK-45* and *AGS* cells in the AR and control groups were determined by wound healing assay. (c) Representative images of the invasion of *MNK-45* and *AGS* cell in the AR and Control groups determined by Transwell invasion assay. (d) Relative invasion rates of *MNK-45* and *AGS* cells in the AR and control groups were determined by Transwell invasion assay. (e) Relative FASN mRNA expression levels in *MNK-45* and *AGS* cells in the AR and control groups were evaluated by quantitative reverse transcription-polymerase chain reaction (qRT-PCR). (f) Representative pictures of relative FASN protein expression levels in *MNK-45* and *AGS* cells in the AR and control groups during western blot. *β*-Actin was used as a loading control. (g) Relative FASN protein expression levels in *MNK-45* and *AGS* cells in the AR and control groups were assessed by western blot. Cells under adherent culture were used as the control group and received no treatment. *^^^^ p* < 0.01 vs. control group; *^^^^^ p* <0.001 vs. control group. Data were performed as the means ± standard.

In the results of colony formation assay, it was presented that the colony numbers of *MNK-45 and AGS* cells in the AR group were greater than those in the control group, indicating that cells with AR were more capable of proliferation (Figures 1(f), 1(g), *p* <0.001). During the wound healing assay and Transwell invasion assay, both the migration and invasion rates of *MNK-45 and AGS* cells were higher in the AR group than in the control group (Figures 2(a)–2(d), *p* < 0.01, *p* <0.001). We also found that the relative mRNA and protein levels of FASN were higher in *MNK-45* and *AGS* cells of the AR group as compared with those of the control group, which suggested an association between FASN and AR (Figures 2(e)–2(g), *p* < 0.01, *p* <0.001).

### 3.3. SiFASN Promoted Apoptosis Yet Suppressed FASN Expression, Aggregation, Migration and Invasion in GC Cells

**Figure 3:**
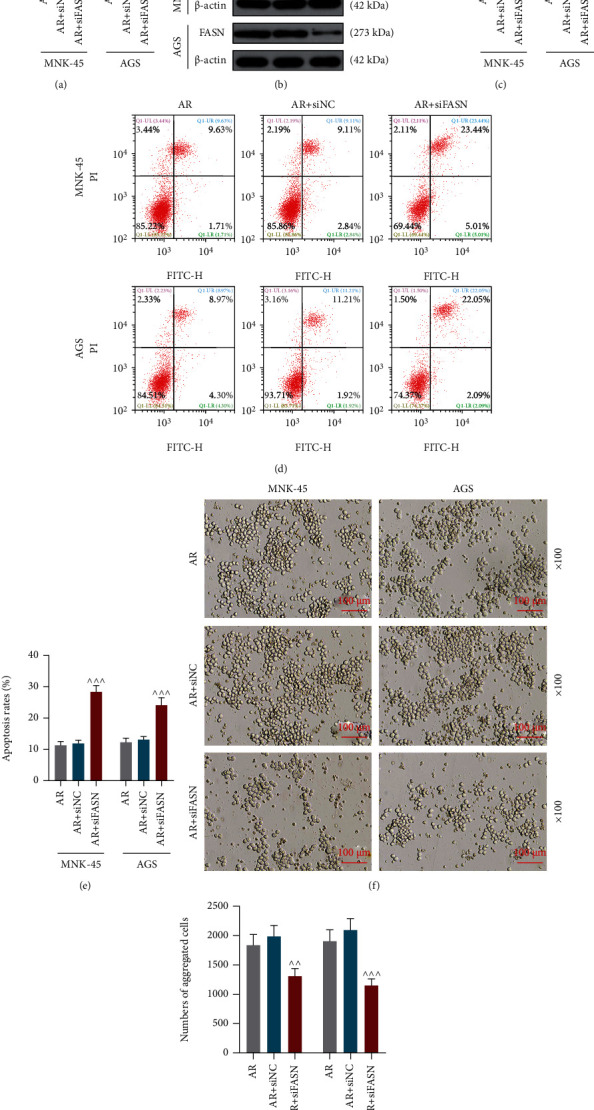
Small interfering RNA targeting fatty acid synthase (siFASN) promoted apoptosis while suppressing cell aggregation and FASN expression. (a) Relative fatty acid synthase (FASN) mRNA expression levels in *MNK-45* and *AGS* cells in the anoikis resistance (AR), AR+ small interfering RNA negative control (siNC), and AR + siFASN groups were evaluated by quantitative reverse transcription-polymerase chain reaction (qRT-PCR). (b) Representative pictures of relative FASN protein expression levels in *MNK-45* and *AGS* cells in the AR, AR + siNC, and AR + siFASN groups during western blot. *β*-Actin was used as a loading control. (c) Relative FASN protein expression levels in *MNK-45* and *AGS* cells in the AR, AR + siNC, and AR + siFASN groups were assessed by western blot. (d) Cell apoptosis was evaluated using a flow cytometer and an Annexin-V-FITC Apoptosis Detection Kit with PI. (e) Quantitation of the apoptosis rates of *MNK-45* and *AGS* cells in the AR, AR + siNC, AR + siFASN groups determined through cell apoptosis assay. (f) Pictures of the morphology of *MNK-45* and *AGS* cells in the AR, AR + siNC, and AR + siFASN groups were taken under a microscope during suspension culture. (g) Numbers of aggregated *MNK-45* and *AGS* cells in the AR, AR + siNC, and AR + siFASN groups were manually counted by microscopy. *^^^^ p* < 0.01 vs. AR + siNC group; *^^^^^ p* <0.001 vs. AR + siNC group. Data were performed as the means ± standard.

**Figure 4:**
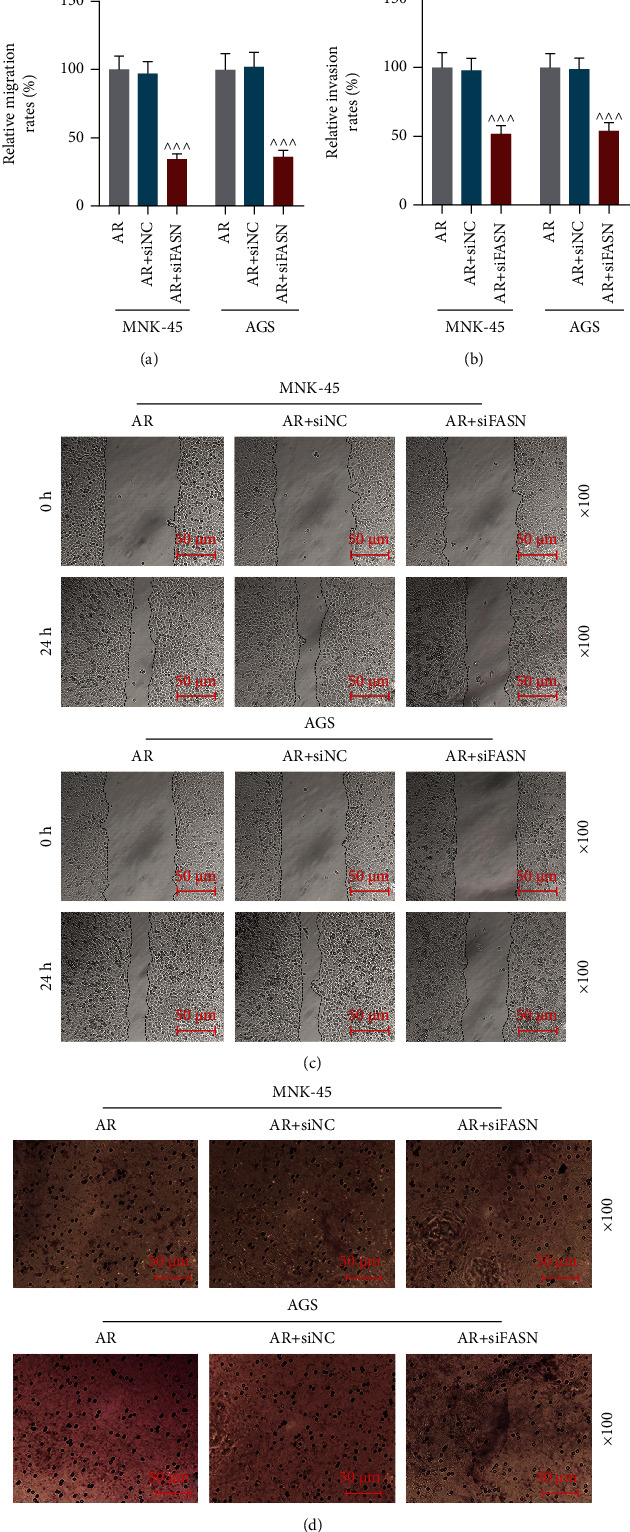
Small interfering RNA targeting fatty acid synthase (siFASN) suppressed cell migration and invasion. (a) Relative migration rates of *MNK-45* and *AGS* cells in the anoikis resistance (AR), AR + small interfering RNA negative control (siNC), and AR + siFASN groups were determined by wound healing assay. (b) Relative invasion rates of *MNK-45* and *AGS* cells in the AR, AR + siNC, and AR + siFASN groups were determined by transwell invasion assay. (c) Representative images of the migration of *MNK-45* and *AGS* cell migration in the AR, AR + siNC, and AR + siFASN groups determined by wound healing assay. (d) Representative images of the invasion of *MNK-45* and *AGS* cell invasion in the AR, AR + siNC, and AR + siFASN groups determined by transwell invasion assay. *^^^^^ p* <0.001 vs. AR + siNC group. Data were performed as the means ± standard.

Both the relative mRNA and protein expression levels of FASN in *MNK-45* and *AGS* cells were lower in the AR + siFASN group than in the AR + siNC group (Figures 3(a)–3(c), *p* <0.001), implying that transfection of siFASN suppressed the expression of FASN. Silence of FASN promoted the apoptosis rates of *MNK-45* and *AGS* cells while decreasing the number of aggregated cells. (Figures 3(d)–3(g), *p* < 0.01, *P* <0.001). In addition, the relative migration and invasion rates of *MNK-45* and *AGS* cells in the AR + siFASN group reduced significantly in comparison with the AR + siNC group (Figures 4(a)–4(d), *p* <0.001).

### 3.4. SiFASN Significantly Suppressed the Activation of P-ERK1/2/Bcl-xL Pathway in GC Cells with AR

**Figure 5:**
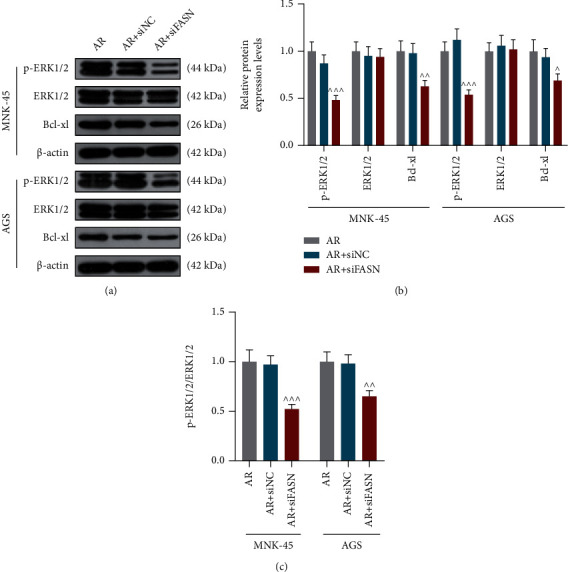
Small interfering RNA targeting fatty acid synthase (siFASN) significantly suppressed p-ERK1/2/Bcl-xL pathway activation in anoikis resistant gastric cancer cells. (a) Representative pictures of the protein levels of phosphorylated (p)-ERK1/2, ERK1/2 and Bcl-xL in *MNK-45* and *AGS* cells in the anoikis resistance (AR), AR + small interfering RNA negative control (siNC), AR + siFASN groups during western blot. *β*-Actin was used as a loading control. (b) Quantitative analysis of p-ERK1/2, ERK1/2 and Bcl-xL expressions normalized to *β*-actin through western blot. (c) The Relative ratio of p-ERK1/2 to ERK1/2 expression. *^^^ p* <0.05 vs. AR + siNC group; *^^^^ p* < 0.01 vs. AR + siNC group; *^^^^^ p* <0.001 vs. AR + siNC. Data were performed as the means ± standard.

The relative protein expression levels of p-ERK1/2 and Bcl-xl in *MNK-45* and *AGS* cells in the AR + siFASN group was detected to be declined while the expression of ERK1/2 was basically unchanged (Figures 5(a), 5(b), *p* < 0.05, *P* < 0.01, *P* <0.001), leading to the lowest ratio of p-ERK1/2 to ERK1/2 in the AR + siFASN group (Figure 5(c), *p*< 0.01, *P* <0.001). These observations suggested that the silence of FASN inhibited the activation of p-ERK1/2/Bcl-xL pathway, and FASN/p-ERK1/2/Bcl-xL pathway might play vital roles in GC with AR.

## 4. Discussion

GC is a kind of deadly malignancy with a low early diagnosis rate and unfavorable prognosis. Tumor metastasis is a major reason for the failure in the treatment and mortality in patients in nearly all types of cancer, and was evidenced in approximately 50% of patients during the development of tumor at initial diagnosis [22, 23]. Cancer metastasis is a complex process comprising different stages, including local invasion, intravasation, survival in circulation, extravasation, and colonization and metastasis, in which a number of interactions between tumor and stroma have been reported [24, 25]. Anoikis, a programmed cell death induced upon the detachment of cells from ECM, is deemed as a crucial mechanism in the preclusion on adherent-independent cell growth and the attachment to an improper matrix, which thereby prevents the metastasis and colonization of cells in distant organs [26]. In addition, Anoikis is one of key procedures to maintain the normal development and homeostasis of organisms [27]. AR, the capability of cells to survive during the detachment from the ECM, is a prerequisite for the development of tumor metastasis [28]. Thus, the competence of cancer cells to resist anoikis has attracted the increasing attention from the scientific community in the field of cancer research, and so far, several mechanisms of AR have been reported and proposed. For instance, integrin-*α*2*β*1/-*α*5*β*1 interacted with epidermal growth factor receptor (EGFR) in the AR of colon cancer [29]. MiR-141/KLF12/Sp1/survivin signaling pathway enhanced AR in ovarian cancer, and a transcriptomic study addressed the importance of CDKN1A-regulated quiescence for cells to survive from anoikis in head and neck squamous carcinoma [30, 31]. In accordance with the discoveries in previous studies, FASN, an enzyme implicated in the process of metabolism, has shown to be positively correlated with the development of various cancers, including GC [32–34]. FASN has also been proved to mediate AR during cell growth and metastasis in osteosarcoma. However, the role of FASN in GC cells with AR has not been explored in detail [19].

In the present study, the observations suggested that AR promoted cell multiplication, migration and invasion and led to high expression levels of FASN in cells, which indicated a possible relation between FASN and AR. We also discovered that silence of FASN significantly reduced cell multiplication, migration and invasion as well as p-ERK1/2 and Bcl-xL levels while barely affecting the expression of ERK1/2. P-ERK1/2 may serve as an important hub in the network, as it occurs crosstalk with other pathways, including phosphatidylinositol 3-kinase (PI3K) [35], and Wnt [36]. Moreover, it has also been demonstrated that there exists a regulatory loop between FASN and ERK pathway, in which PI3K/AKT pathway is involved as well [37]. Therefore, the regulation between FASN and ERK pathway may be involved with other molecular pathways. PI3K/AKT pathway also regulates downstream anti-apoptotic proteins, including Bcl-xL [38]. Thus, it was inferred that the functions of FASN on GC cells with AR might be related to p-ERK1/2/Bcl-xL pathway, regardless of other molecular pathways that may be involved.

However, there is room for improvement of the current study. Firstly, the length of FASN mRNA is about 8.4 kb, making the clone on the full length a great challenge. Thus, cell lines with overexpressed FASN failed to be established, leaving the results and conclusions with one-sided uncertainty. Also, the specific molecular mechanism of FASN in GC cells with AR was not absolutely settled. Secondly, the experiments for detecting cell apoptosis rates and colony formation could be conducted more scrupulously by the exclusion of the influence of cell proliferation. Thirdly, we directly selected p-ERK1/2/Bcl-xL based on documents and assumption, and this blindness could be avoided by probing into the downstream signaling pathways of FASN. Lastly, researches *in vivo* should be carried out in the future to further prove the conclusions of experiments *in vitro*.

In summary, this study demonstrated the stronger AR of GC cells, which may explain the survival of cells during the metastasis and spread of GC. The results revealed that AR facilitated multiplication, migration and invasion while impeding apoptosis of GC cell. Moreover, it was preliminarily verified that the down-regulation of FASN exerted suppressive effects on GC cells with AR, which was associated with the inhibition of p-ERK1/2/Bcl-xL signaling pathway. Taken together, our results and data indicate that FASN may become a new target for preventing the metastasis of tumor, thus providing a novel reference for the treatment against cancer. Nonetheless, more researches and clinical trials are needed to explore whether FASN is a feasible mean for the prevention and treatment of GC.

## Figures and Tables

**Table 1:** quantitative reverse transcription-polymerase chain reaction (qRT-PCR) primers.

Target gene	Forward primers, 5'-3'	Reverse primers, 5'-3'
*β*-Actin	CTCCATCCTGGCCTCGCTGT	GCTGTCACCTTCACCGTTCC
FASN	CAACTCACGCTCCGGAAA	TGTGGATGCTGTCAAGGG

## Data Availability

The analyzed data sets generated during the study are available from the corresponding author on reasonable request.
